# Clinical and functional analyses of *AIPL1* variants reveal mechanisms of pathogenicity linked to different forms of retinal degeneration

**DOI:** 10.1038/s41598-020-74516-9

**Published:** 2020-10-16

**Authors:** Almudena Sacristan-Reviriego, Hoang Mai Le, Michalis Georgiou, Isabelle Meunier, Beatrice Bocquet, Anne-Françoise Roux, Chrisostomos Prodromou, James Bainbridge, Michel Michaelides, Jacqueline van der Spuy

**Affiliations:** 1 UCL Institute of Ophthalmology, University College London, 11 – 43 Bath Street, London, EC1V 9EL UK; 2 Moorfields Eye Hospital, City Road, London, EC1V 2PD UK; 3 Centre National de Référence Maladies Sensorielles Génétiques, Service Ophtalmologie Hôpital Gui de Chauliac - CHRU de Montpellier, 80 rue Augustin Fliche, 34295 Montpellier, France; 4 Laboratoire de Génétique Moléculaire, CHU de Montpellier, Université de Montpellier, Montpellier, France; 5 Biochemistry and Biomedicine, University of Sussex, Brighton, Falmer, BN1 9QG UK

**Keywords:** Cell biology, Molecular biology, Diseases, Medical research, Pathogenesis

## Abstract

Disease-causing sequence variants in the highly polymorphic *AIPL1* gene are associated with a broad spectrum of inherited retinal diseases ranging from severe autosomal recessive Leber congenital amaurosis to later onset retinitis pigmentosa. AIPL1 is a photoreceptor-specific co-chaperone that interacts with HSP90 to facilitate the stable assembly of retinal cGMP phosphodiesterase, PDE6. In this report, we establish unequivocal correlations between patient clinical phenotypes and in vitro functional assays of uncharacterized AIPL1 variants. We confirm that missense and nonsense variants in the FKBP-like and tetratricopeptide repeat domains of AIPL1 lead to the loss of both HSP90 interaction and PDE6 activity, confirming these variants cause LCA. In contrast, we report the association of p.G122R with milder forms of retinal degeneration, and show that while p.G122R had no effect on HSP90 binding, the modulation of PDE6 cGMP levels was impaired. The clinical history of these patients together with our functional assays suggest that the p.G122R variant is a rare hypomorphic allele with a later disease onset, amenable to therapeutic intervention. Finally, we report the primate-specific proline-rich domain to be dispensable for both HSP90 interaction and PDE6 activity. We conclude that variants investigated in this domain do not cause disease, with the exception of p.A352_P355del associated with autosomal dominant cone-rod dystrophy.

## Introduction

The aryl hydrocarbon receptor interacting protein-like 1 (AIPL1) is a retinal photoreceptor-specific protein expressed in cones and rods^1,2^. AIPL1 acts, in concert with HSP90, as a specialized molecular co-chaperone for cGMP-specific phosphodiesterase-6 (PDE6), a key effector of the visual phototransduction cascade^3–6^. AIPL1 facilitates the stability and catalytic activity of heterologously expressed cone and rod PDE6^3,5^. Studies in mice have shown that in the absence of AIPL1, the levels of cone and rod PDE6 are markedly reduced^7–9^ and the PDE6 subunits are misassembled and targeted to proteasomes for degradation^10^. The increased levels of cGMP trigger rapid degeneration of photoreceptors and, as a result, electroretinography (ERG) responses are extinguished at any age tested in the absence of AIPL1^9,11^. Interestingly, disease-causing sequence variants in *PDE6* also cause increased levels of cGMP and subsequent retinal degeneration^12–16^.

The 384-amino acid protein AIPL1 is composed of an N-terminal FK506-binding protein (FKBP)-like domain, a tetratricopeptide repeat (TPR) domain and a primate-specific C-terminal proline rich domain (PRD). The TPR domain, comprising three TPR motifs, is responsible for the interaction of AIPL1 with HSP90, which is facilitated by the TPR-acceptor site EEVD located at the C-terminus of HSP90^4,5^. The N-terminal FKBP-like domain of AIPL1 facilitates the interaction with the C-terminal isoprenyl (farnesyl and geranylgeranyl) moieties present in PDE6 subunits due to posttranslational modifications^17–20^. Thus, variants compromising the integrity and relative orientation of the AIPL1 FKBP-like or TPR domain fail to efficiently modulate PDE6 activity in vitro through HSP90^5^.

Biallelic variants in the *AIPL1* gene are predominantly associated with autosomal recessive Leber congenital amaurosis (LCA), a congenital-onset, rapid and progressive retinal degeneration leading to the severe impairment or loss of vision within the first few years of life^21–24^. Interestingly, there are reports of less severe later onset retinal degeneration diagnoses such as retinitis pigmentosa (RP) linked to biallelic *AIPL1* variants^25^. Moreover, a heterozygous 12-bp deletion leading to a protein lacking four amino acids in the PRD has been reported to be a cause of autosomal dominant cone-rod dystrophy (adCORD) and early-onset RP^26^.

LCA-associated *AIPL1* sequence variants to date include missense and nonsense variants, as well as small insertions and duplications, small deletions and splice alterations. Interestingly, missense variants in the very first or last nucleotides of exons, as well as small insertions or duplications spanning intron–exon boundaries, have been shown to induce aberrant pre-mRNA *AIPL1* splicing, thereby resolving the disease-causing status and pathogenic mechanisms for these sequence variants^27^. Presently, the Human Gene Mutation Database (HGMD)^28^, the ClinVar database^29^ and the Ensembl project^30^ have recorded 83, 125 and 32 *AIPL1* variants respectively. Several of these are considered likely to be benign or rare ethnic benign single nucleotide polymorphisms, while several other variants are still of uncertain or conflicting pathogenicity. Confirmation of true disease-associated *AIPL1* variants is therefore a challenge confounded by the polymorphic nature of the gene and remains crucial for patient diagnosis and potential *AIPL1*-targeted gene replacement therapies^31^.

In this study, we determined the disease-causing status of novel and previously reported but uncharacterized AIPL1 variants, and established a correlation between clinical phenotypes and the underlying pathogenic mechanisms through in vitro functional assays. We confirm the disease-causing status of several variants investigated and the possibility of rare hypomorphic alleles, thus clarifying the prospects for therapeutic intervention for patients harbouring these variants.

## Results

### Molecular genetics

In this study, a total of 21 *AIPL1* sequence variants were investigated, including missense (n = 14) and nonsense (n = 3) variants, as well as small insertions (n = 1), duplications (n = 2) and deletions (n = 1) in the coding sequence (Tables 1 and 2; full clinical details in Suppl. Table 1). These variants have been identified in patients in previously reported studies or this study. Ten of the sequence variants investigated have been identified in a homozygous or compound heterozygous state in patients with molecularly confirmed biallelic variants in *AIPL1* (Table 1, Suppl. Table 1). Thirteen sequence variants, 2 of which were also reported in patients with biallelic variants, have been identified on only a single allele in patients with no change identified on the other allele (Table 2, Suppl. Table 1).

**Table 1:** Clinical information of patients with *AIPL1* variations: Patients with *AIPL1* variations identified on both alleles.

Case	Allele 1	Allele 2	Clinical diagnosis	References
**P1**	c.116C>A; **p.T39N**	c.116C>A; **p.T39N**	LCA	^32^
**P2**	c.214T>C; **p.W72R**	c.265T>C; p.C89R	LCA	^25^
**P3**	c.266G>A; **p.C89Y**	c.266G>A; **p.C89Y**	LCA	^33^
**P4**	c.364G>A; **p.G122R**	c.834G>A; p.W278X	LCA	^34^
**P5**	c.364G>A; **p.G122R**	c.364G>A; **p.G122R**	RP	This study
**P6**	c.364G>C; **p.G122R**	c.834G>A; p.W278X	RP/late onset retinal degeneration	^25^
**P7**	c.364G>C; **p.G122R**	c.834G>A; p.W278X	Mild RP	This study
**P8**	c.364G>C; **p.G122R**	c.834G>A; p.W278X	Mild RP	This study
**P9**	c.582C>G; **p.Y194X**	c.834G>A; p.W278X	LCA	^35^
P10*	c.666G>A; p.W222X*	c.834G>A; p.W278X	LCA	This study
**P11**	c.733G>T; **p.E245X**	c.834G>A; p.W278X	LCA	^35^
**P12**	c.809G>A; **p.R270H**	c.834G>A; p.W278X	LCA	^36^
**P13**	c.809G>A; **p.R270H**	c.834G>A; p.W278X	LCA	^34^
**P14**	c.809G>A; **p.R270H**	c.834G>A; p.W278X	LCA	^33^
**P15**	c.926_927ins CCTGAACCGCAGGGAGCT; **p.E309DinsLNRREL**	c.926_927ins CCTGAACCGCAGGGAGCT; **p.E309DInsLNRREL**	LCA	^37^
**P16**	c.1126C>T; **p.P376S**	c.341C>T; p.T114I	LCA	^26^
**P17**	c.1126C>T; **p.P376S**	c.341C>T; p.T114I	LCA	^23^
**P18**	c.1126C>T; **p.P376S**	c.341C>T; p.T114I	LCA	^23^
**P19**	c.1126C>T; **p.P376S**	c.341C>T; p.T114I	EOSRD LCA	^38^
**P20**	c.1126C>T; **p.P376S**	c.341C>T; p.T114I	EOSRD	^39^ This study
**P21**	c.1126C>T; **p.P376S**	c.1126C>T**; p.P376S**	EOSRD	^39^ This study

The AIPL1 variants investigated in this study are shown in **BOLD**. LCA, Leber congenital amaurosis; EOSRD, early-onset severe retinal dystrophy; RP, retinitis pigmentosa; *, novel variation.

**Table 2:** Clinical information of patients with *AIPL1* variations: Patients with a single *AIPL1* variation identified on one allele.

Case	Allele 1	Allele 2	Clinical diagnosis	References
**P22**	c.157C>T; **p.R53W**	N/A	LCA	^40^
**P23**	c.214T>C; **p.W72R**	N/A	LCA	^41^
**P24**	c.390C>A; **p.H130Q**	N/A	EOSRD LCA	^39^
**P25**	c.593C>T; **p.S198F**	N/A	EOSRD LCA	^39^
**P26**	c.617T>A; **p.I206N**	N/A	LCA	^40^
**P27**	c.878T>C, **p.L293P**	N/A	LCA	^41^
**P28**	c.894G>C; **p.Q298H**	N/A	EOSRD LCA	^39^
**P29**	c.1053_1064del TGCAGAGCCACC; **p.A352_P355del**	N/A	adCORD, juvenile RP	^26^
**P30**	c.1091C>G; **p.A364G**	N/A	EOSRD LCA	^39^
**P31**	c.1097C>G; **p.P366R**	N/A	EOSRD LCA	^39^
**P32**	c.1103_1114dup; **p.E369_T372dup**	N/A	LCA	^41^
**P33**	c.1111_1122dup; **p.A371_P374dup**	N/A	LCA	^42^
P34*	c.1126C>T; **p.P376S**	N/A	LCA	^41^
**P35**	c.1126C>T; **p.P376S** c.341C>T; p.T114I *Cis*-allelic inheritance from mother	N/A	LCA	^43^
**P36**	c.1126C>T; **p.P376S**	N/A	EOSRD	^39^ This study
**P37**	c.1126C>T; **p.P376S**	N/A	RP EOSRD	^39^ This study

The AIPL1 variants investigated in this study are shown in **BOLD**. LCA, Leber congenital amaurosis; EOSRD, early-onset severe retinal dystrophy; RP, retinitis pigmentosa; adCORD; autosomal dominant cone-rod dystrophy; N/A, not available;* , did not segregate with disease.

### Clinical phenotype of AIPL1-associated cases

#### Patients with biallelic AIPL1 sequence variants

**Figure 1:**
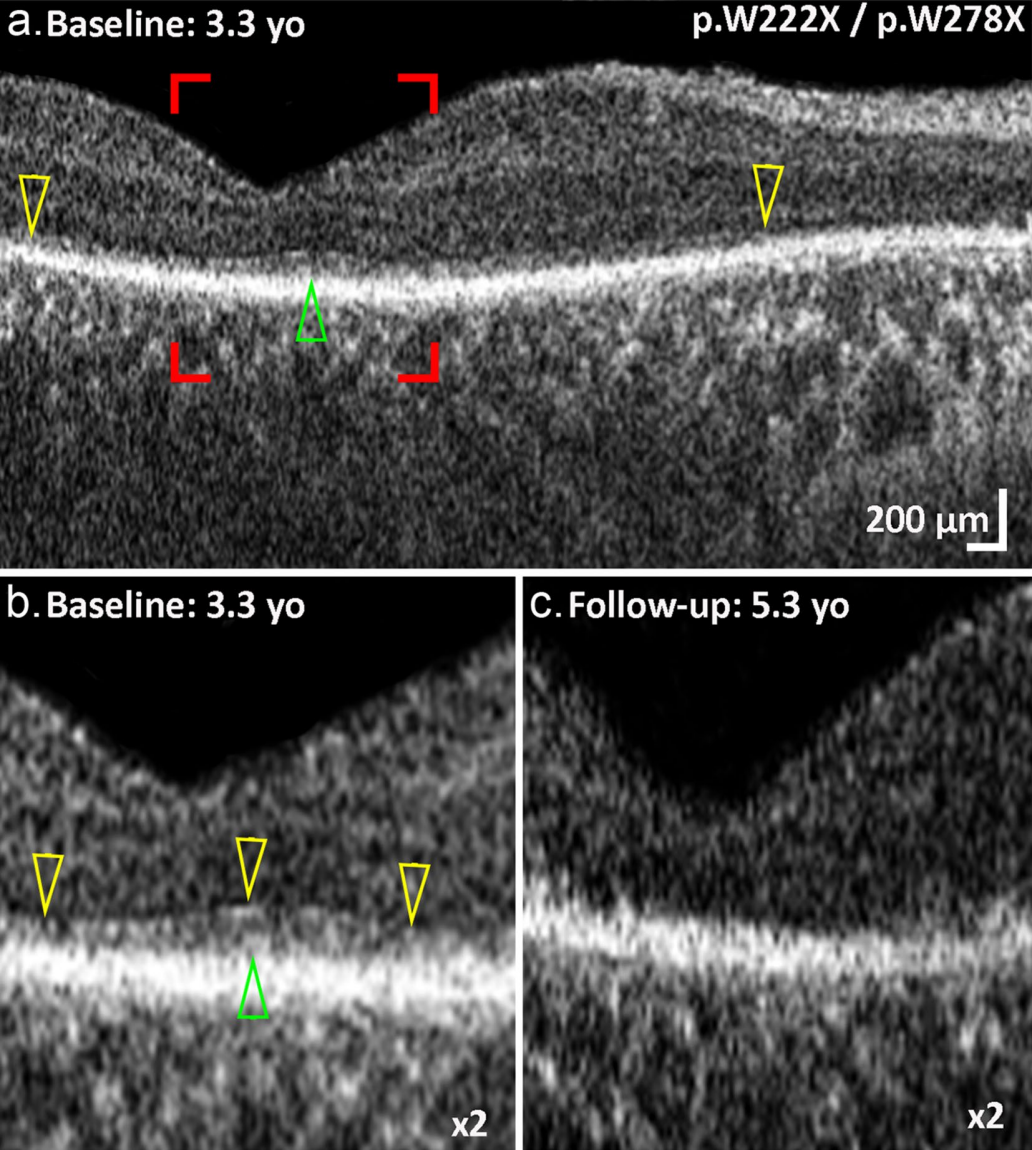
Longitudinal optical coherence tomography (OCT) imaging in an LCA patient with *AIPL1* c.666G>A (p.W222X)/c.834G>A (p.W278X) variants. (**a**) Transfoveal OCT image of the right eye at baseline. The yellow arrowheads mark the temporal and nasal borders of the external limiting membrane (ELM). There is extensive loss of photoreceptors and loss of the ellipsoid zone (EZ). The green arrowhead marks the residual EZ over the foveal center. The red borders mark the region of interest shown at higher magnification (× 2) in (**b**), where the ELM is marked with yellow arrowheads and the remnants of photoreceptors with a green arrowhead. After 2 years follow-up (**c**), no ELM or EZ remnants were identified and the retinal thickness was decreased compared to (**b**). Visual acuity was hand motion at 30 cm at baseline and perception of light at follow-up. yo; years old.

The majority of patients with biallelic *AIPL1* variants were diagnosed with LCA (Table 1, Suppl. Table 1). These patients have severely reduced visual acuity, ranging from 20/400 to perception of light (PL), variable pigmentary changes and macular atrophy on fundoscopy, and undetectable or markedly diminished rod and cone ERGs (Suppl. Table 1). P10 was compound heterozygous for c.834G>A (p.W278X) and a novel *AIPL1* variant c.666G>A (p.W222X) identified in this study. This patient was diagnosed with LCA, with congenital onset and complete loss of the neurosensory retina by the age of five years (Fig. 1).

P19, P20 and P21 were diagnosed with early onset severe retinal dystrophy (EOSRD), with P20 having milder disease compared to P21 (no clinical details available for P19). The c.1126C>T (p.P376S) sequence variant was detected in each of these patients either homozygously (P21) or heterozygously together with c.341C>T (p.T114I) (P19, P21). The VA in these patients ranged from 6/19 to PL (Suppl. Table 1).

**Figure 2:**
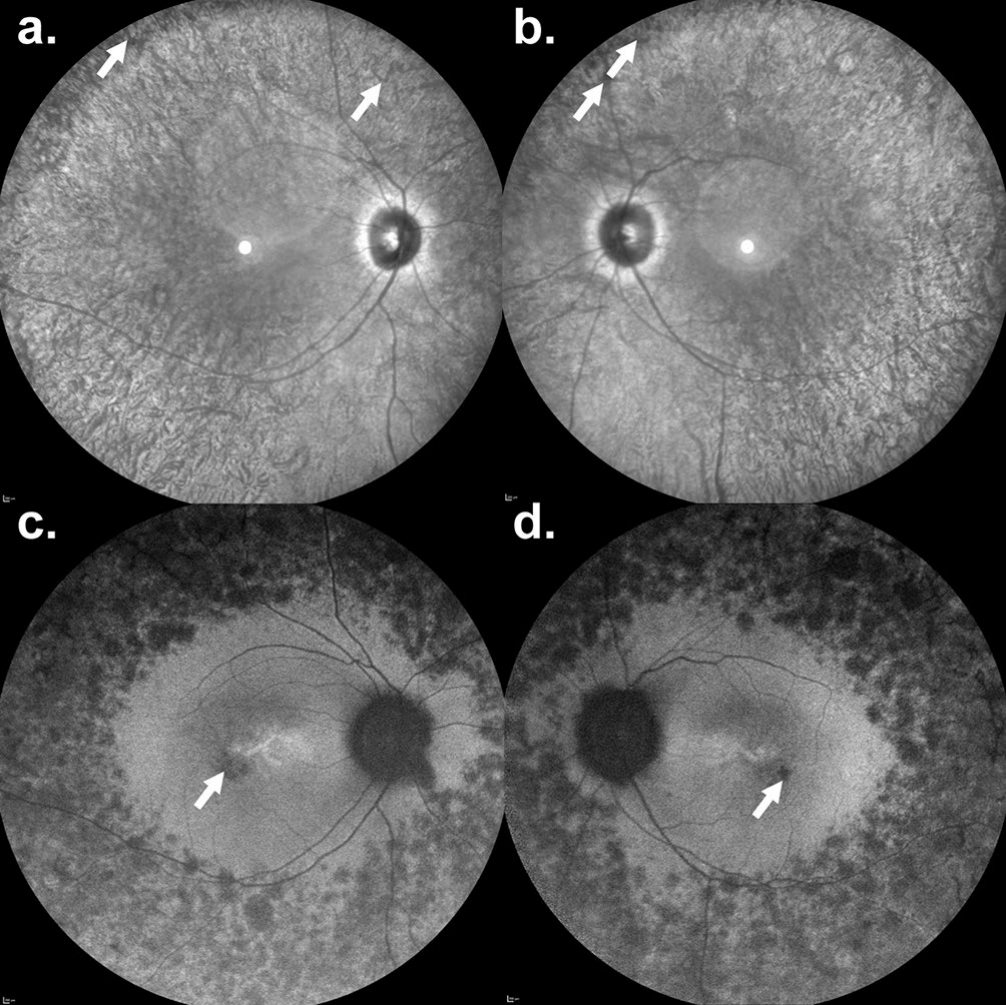
A 34-year-old man with a retinitis pigmentosa phenotype carrying a homozygous *AIPL1* c.364G>A (p.G122R) variant. Nyctalopia since early childhood was described by the patient. Visual acuity was 20/50 in the right eye and 20/100 in the left eye. (**a**,**b**) Infrared reflectance imaging. The retinal vessel diameters are reduced with bone-spicule-like pigmentation in the peripheral retina (white arrows). Fundus autofluorescence revealed multiple hypoautofluorescent spots within the peripheral retina. There was no macular ring of increased signal, but small hypoautofluorescent lesions were seen within the fovea (white arrows).

**Figure 3:**
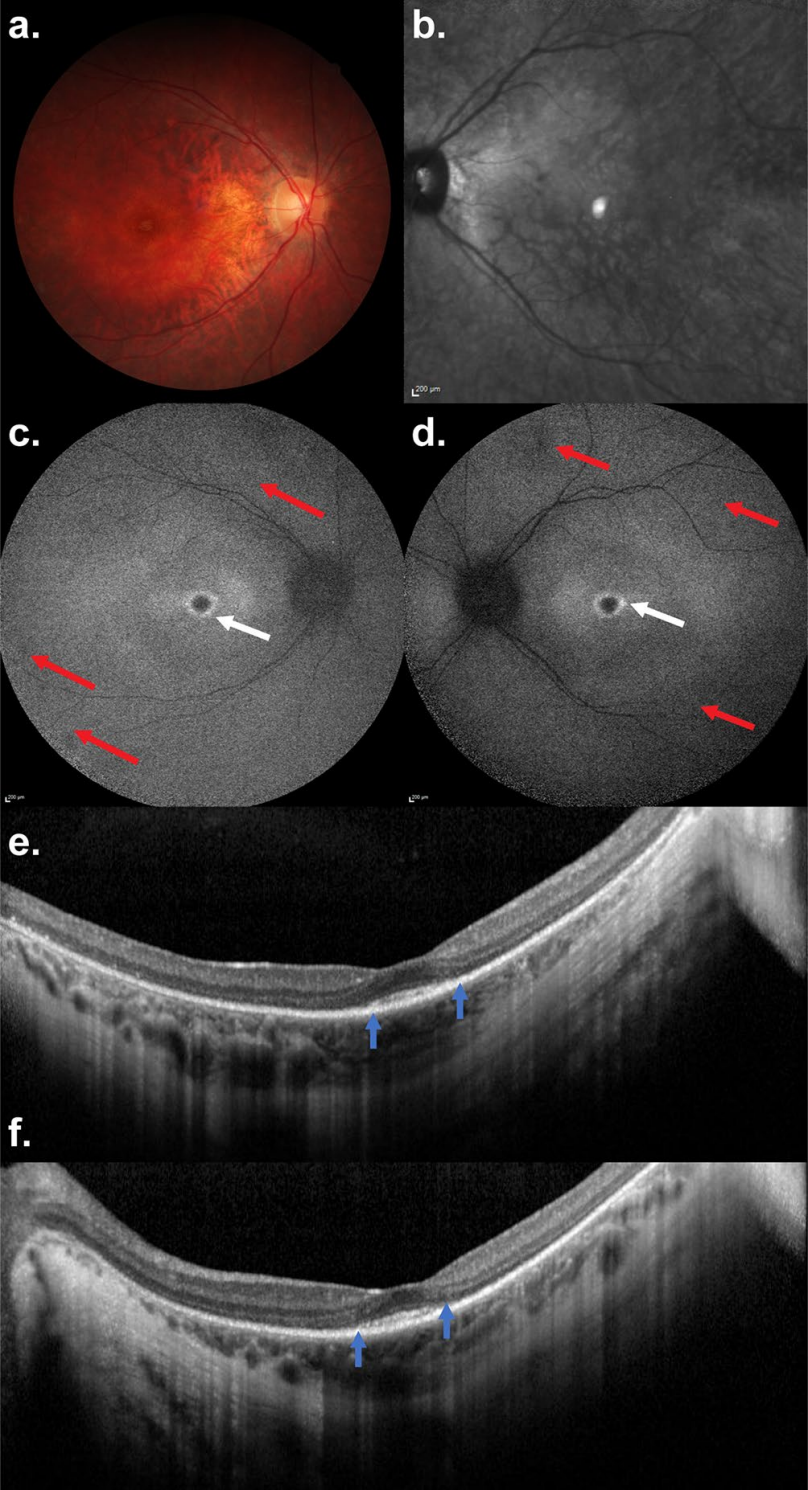
An 18-year-old woman with a retinitis pigmentosa phenotype with *AIPL1* c.364G>C (p.G122R)/c.834G>A (p.W278X) variants. Nyctalopia was the first symptom, noted at the age of 5 years. Visual acuity was 20/20 in both eyes and the patient did not report peripheral or central visual field loss. (**a**) Colour fundus photography in the right eye and (**b**) reflectance infrared imaging of the left eye: note mild reduced diameter of the retinal vessels and the lack of bone-spicule-like pigmentation in the peripheral retina. (**c**,**d**) Fundus autofluorescence imaging disclosed a macular hyperautofluorescent ring (white arrows) and few peripheral hypoautofluorescent spots (red arrows). (**e**,**f**) On SD-OCT, there was extensive loss of the photoreceptor/retinal pigment epithelium complex and the ellipsoid zone (EZ). EZ and the interdigitation zone are only visible at the fovea (between blue arrows) in accordance with the visual acuity of 20/20.

P5-P8 did not have a phenotype within the LCA/EOSRD spectrum. P5 was homozygous for a c.364G>A (p.G122R) sequence variant and has RP (this study) (Fig. 2, Suppl. Fig. 1). The patient presented with nyctalopia since early childhood with a VA at 34 years old of 20/50 in the right eye and 20/100 in the left eye. Interestingly, P4 is compound heterozygous for the same c.364G>A (p.G122R) variant on one allele and the common disease-causing variant c.834G>A (p.W278X) on the other allele, but was diagnosed with LCA^34^. P6-P8 were all diagnosed with late onset retinal degeneration or RP and all harbour a different missense variant coding for the same amino acid change (c.364G>C (p.G122R)) as P4 and P5 on one allele, together with the c.834G>A (p.W278X) sequence variant on the other allele. P6 is a previously reported case with severely decreased VA (20/60, 20/400), undetectable ERG and a diagnosis of late onset retinal degeneration or RP^25^. P7 (Fig. 3, Suppl. Fig. 1) and P8 (Suppl. Fig. 2) are siblings and presented with nyctalopia and mild signs of RP with unaffected visual acuity (20/20) (this study).

#### Patients with monoallelic AIPL1 sequence variants

Thirteen heterozygous *AIPL1* sequence variants previously reported in patients with retinal degeneration but without detailed phenotyping and with no *AIPL1* changes identified in the coding regions or intron/exon boundaries of the other allele were also investigated (Table 2, Suppl. Table 1)*.* Two of these sequence variants, c.214T>C (p.W72R) and c.1126C>T (p.P376S), were also found in patients with biallelic *AIPL1* variants (see Table 1: Patients with *AIPL1* variations identified on both alleles). All available clinical features are summarised in Suppl. Table 1.

The majority of patients were diagnosed with LCA/EOSRD. The sequence variant c.1053_1064del12pb (p.A352_P355del) was previously reported to cause adCORD or early-onset RP^26^ (Table 2, Suppl. Table 1). P36 and P37, harbouring a heterozygous c.1126C>T (p.P376S) change, were diagnosed with EOSRD or EOSRD/RP respectively. Interestingly, a c.1126C>T (p.P376S) heterozygous change was previously reported not to segregate with disease^41^ and Coppieters et al.^43^ reported the maternal *cis*-allelic inheritance of c.1126C>T (p.P376S) and c.341C>T (p.T114I) (Table 2, Suppl. Table 1).

### In silico analysis and allele frequencies of AIPL1 variants

The in silico analysis of the *AIPL1* variants resulting from missense changes are summarized in Table 3. Most of the variants investigated in this study fall in the category of damaging according to both the SIFT tolerance index and Polyphen HumVar Score with the exception of the p.A364G variant, which is scored as tolerated/benign. There is a discrepancy in the results obtained for p.Q298H and p.P376S variants, which are scored as tolerated by one program and damaging by the other. Analysis of the allele frequency of *AIPL1* missense and nonsense sequence variations show that all the *AIPL1* variants investigated are very rare (allele frequency < 1/10,000) and have not been observed in the homozygous state in the current Exome Aggregation Consortium (ExAC) and the Genome Aggregation Database (gnomAD) with the exception of c.1126C>T (p.P376S), detected in 27 and 51 homozygotes respectively, and a frequency of 59–62 per 10,000.

**Table 3:** Allele frequencies and in silico predictions of the AIPL1 variants investigated.

Nucleotide change	Protein change	SIFT	Polyphen	Allele frequency		Number of Homozygotes	
				ExAC	gnomAD	ExAC	gnomAD
c.116C>A	p.T39N	D 0.05	P.D 1	nd	nd	nd	nd
c.157C>T	p.R53W	D 0	P.D 0.96	0.000008358	0.00004246	0	0
c.214T>C	p.W72R	D 0	P.D 1	nd	nd	nd	nd
c.266G>A	p.C89Y	D 0	P.D 1	nd	0.000003996	nd	0
c.364G>A	p.G122R	D 0	P.D 1	0.00000834	0.000003988	0	0
c.390C>A	p.H130Q	D 0	P.D 0.998	nd	0.000003992	nd	0
c.582C>G	p.Y194X	N/A	N/A	0.000008238	0.000003976	0	0
c.593C>T	p.S198F	D 0.01	p.D 0.627	nd	nd	nd	nd
c.617T>A	p.I206N	D 0.03	P.D 0.953	nd	nd	nd	nd
c.666G>A	p.W222X	N/A	N/A	nd	nd	nd	nd
c.733G>T	p.E245X	N/A	N/A	nd	nd	nd	nd
c.809G>A	p.R270H	D 0	P.D 1	nd	nd	nd	nd
c.878T>C	p.L293P	D 0	P.D 1	nd	nd	nd	nd
c.894G>C	p.Q298H	T 0.11	p.D 0.721	nd	nd	nd	nd
c.1091C>G	p.A364G	T 0.1	Benign 0.001	nd	nd	nd	nd
c.1097C>G	p.P366R	D 0.02	p.D 0.637	nd	nd	nd	nd
c.1126C>T	p.P376S	D 0	Benign 0.001	0.00588	0.006198	27	51

The cDNA is numbered according to the longest AIPL1 transcript ENST00000381129, protein ID ENSP00000370521. ExAC allele count: Allele frequency of the *AIPL1* variations in a reference data set derived from 61,486 unrelated individuals sequenced as part of various disease-specific and population genetic studies; excludes cases of severe pediatric disease. The dataset provided in gnomAD includes 125,748 exome sequences and 15,708 whole-genome sequences from unrelated individuals sequenced as part of various disease-specific and population genetic studies. SIFT results: tolerance index ≥ 0.05 = tolerated (T), tolerance index < 0.05 = damaging (D). PolyPhen-2: benign, possibly damaging (p.D), probably damaging (P.D). N/A, not applicable; nd, not described.

### Expression and subcellular distribution of AIPL1 variants

**Figure 4:**
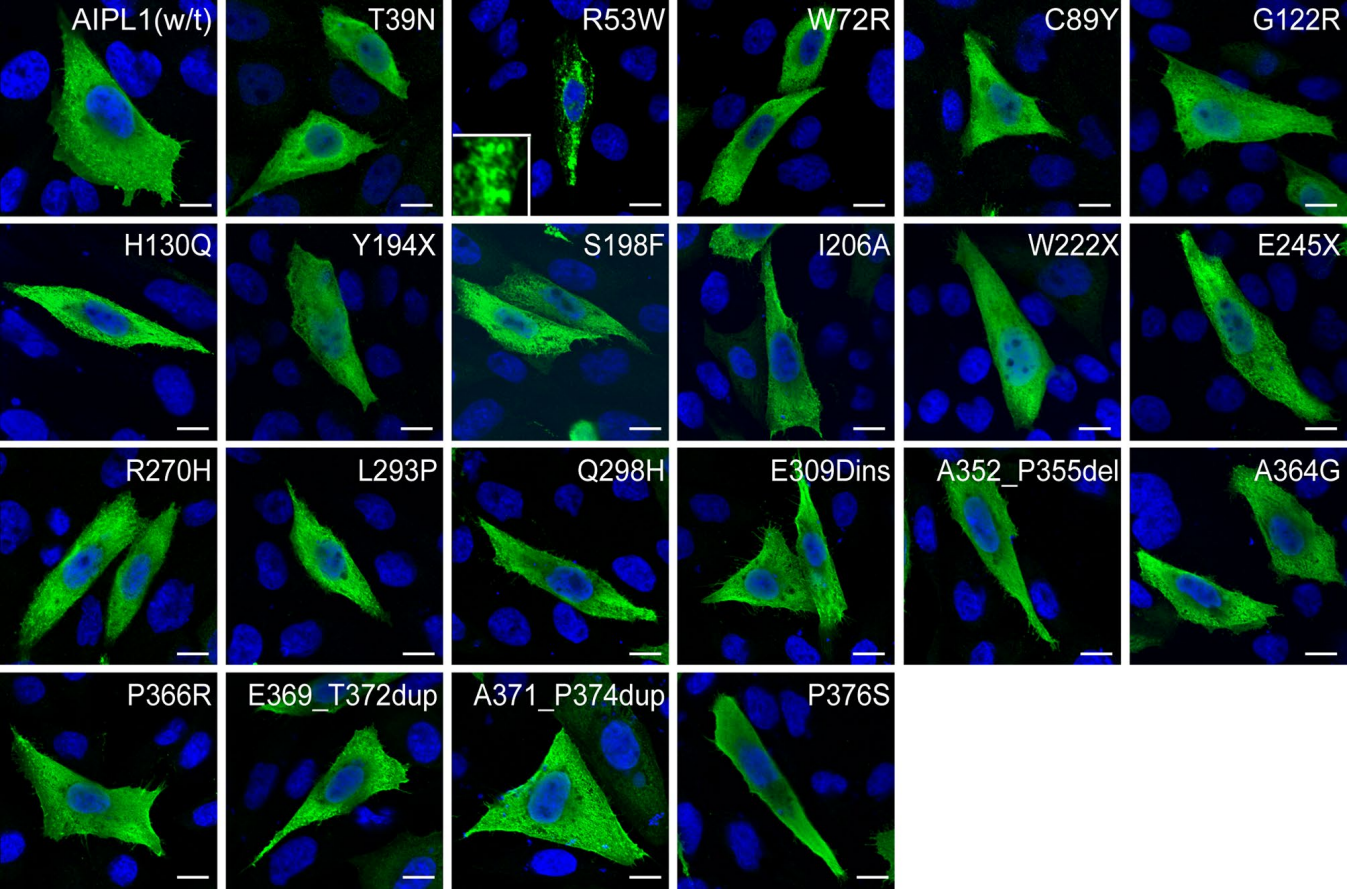
Subcellular localization of AIPL1 variants. Indirect immunofluorescent confocal microscopy using anti-myc antibody against the N-terminus of AIPL1. The misfolding and aggregation of p.R53W to visible intracellular inclusions is shown at higher magnification as an insert. Scale bar: 10 μm.

**Figure 5:**
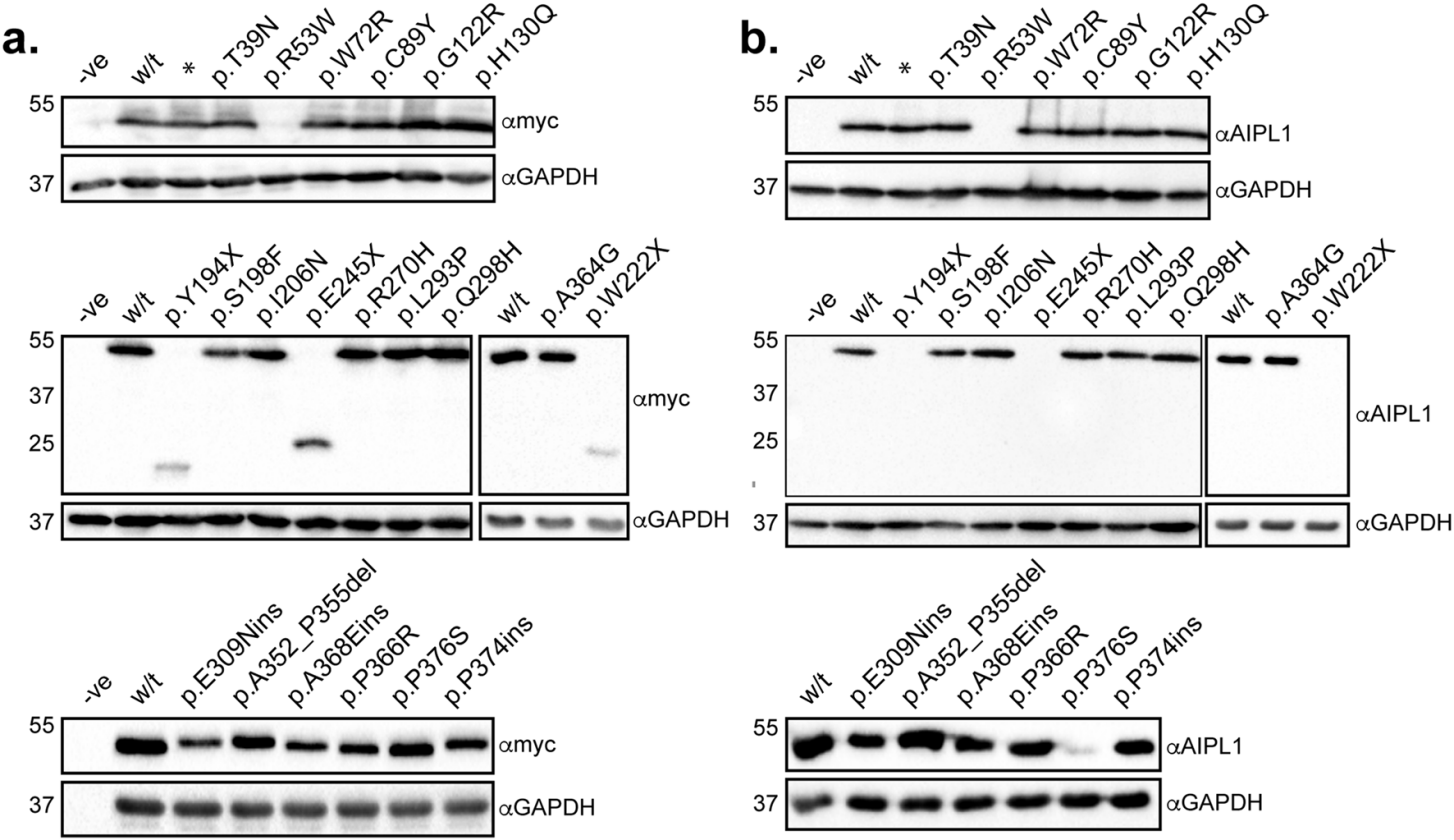
Expression of AIPL1 variants. Western blotting analysis of wild-type (w/t) AIPL1 and the indicated AIPL1 variants. The proteins were resolved by SDS-PAGE on a 12% gel. (**a**) The anti-myc antibody recognizes the myc tag fused to the N-terminus of AIPL1. (**b**) The anti-AIPL1 antibody is directed against a C-terminal AIPL1 epitope^1^. GAPDH was detected as a loading control. *Demarcates an in silico designed AIPL1 variant not found in patients and not associated with disease.

We first examined the expression and subcellular localization of the AIPL1 variants described above by immunofluorescent confocal microscopy (Fig. 4) and western blotting (Fig. 5). The myc-tagged AIPL1 variants p.T39N, p.W72R, p.C89Y, p.G122R, p.H130Q, p.Y194X, p.S198F, p.I206N, p.W222X, p.E245X, p.R270H, p.L293P, p.Q298H, p.A364G, p.E309Dins, p.A352_P355del, p.E369_T372dup, p.P366R, p.A371_P374dup and p.P376S showed a homogeneous subcellular cytoplasmic distribution similar to wildtype (w/t) AIPL1 (Fig. 4). The p.R53W variant formed intracellular inclusions (Fig. 4) and was not detected by immunoblotting (Fig. 5), pointing to the misfolding of the p.R53W variant to detergent insoluble aggregates that could not be resolved by denaturing SDS-PAGE. This mutation drastically alters the charge at a highly conserved residue within a single turn α helix (αt) between the β3 and β4 strands of the FKBP-like domain^19^, thus most likely having a detrimental impact on the folding of this domain.

The analysis of expression by western blotting with an anti-myc antibody directed against the N-terminal myc tag revealed that the levels of expression of most of the AIPL1 variants investigated were similar to w/t AIPL1 in the soluble fraction except for the nonsense variants p.Y194X, p.W222X and p.E245X, whose levels were decreased (Fig. 5a). These variants, resulting in a large C-terminal truncation, were not detected by western blotting with the anti-hAIPL1 antibody directed against the C-terminal AIPL1-specific epitope (Fig. 5b). Reduced levels of the variant p.P376S were detected with the C-terminal anti-hAIPL1 antibody, most likely due to disruption of the anti-hAIPL1 epitope^1^. Full blots and relative quantitation of the levels are shown in Suppl. Fig. 3.

### Analysis of AIPL1 function

**Figure 6:**
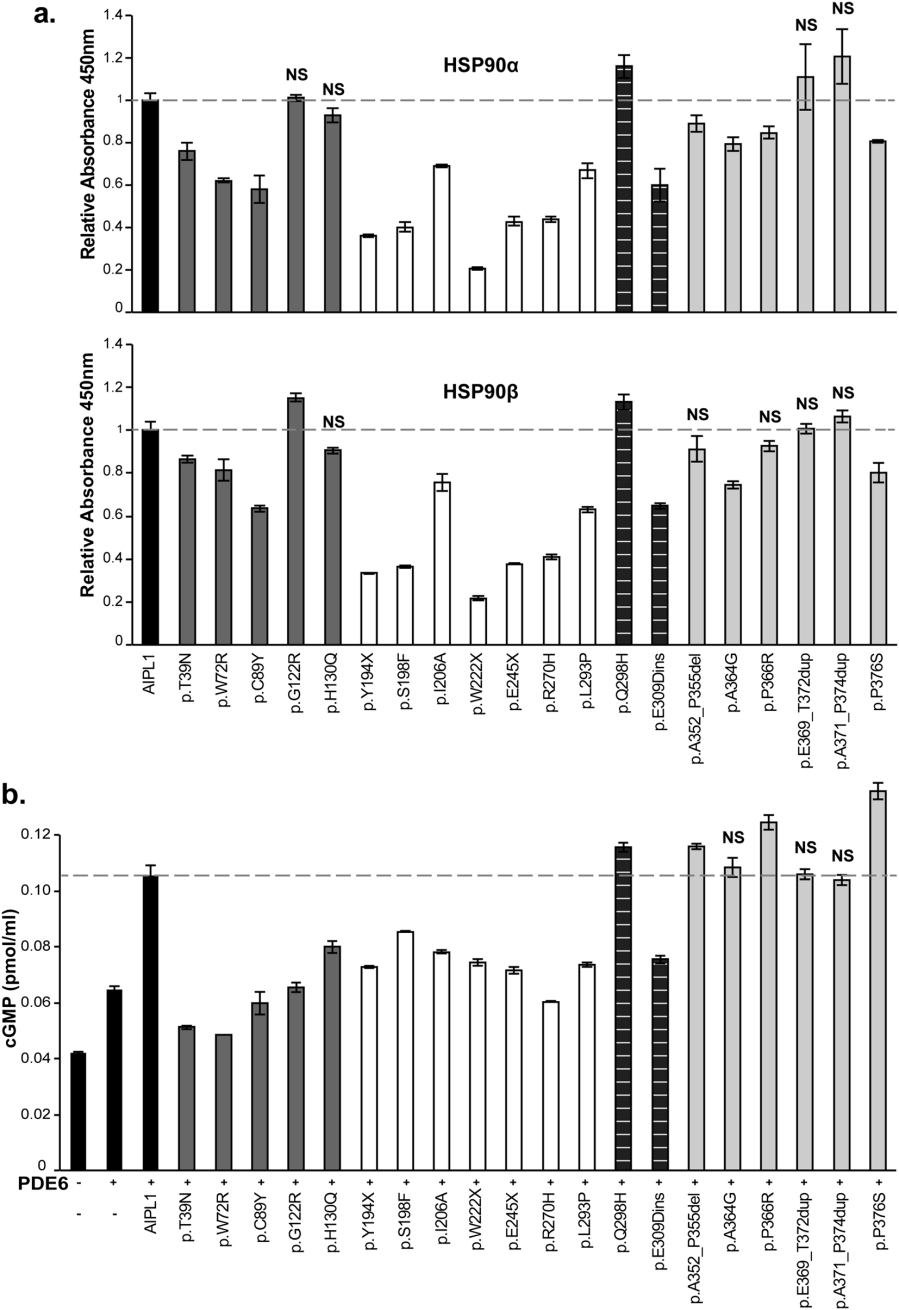
Functional analysis of AIPL1 variants. (**a**) Interaction of AIPL1 variants with HSP90. Quantitative ELISA analysis of the interaction between wild-type (w/t) AIPL1 or the AIPL1 variants indicated with purified human recombinant HSP90α and HSP90β. The absorbance of each interaction was normalized to the expression level detected by western-blotting and plotted relative to w/t AIPL1 = 1.0. (**b**) Heterologous rod PDE6 activity in the presence of the AIPL1 variants. Quantitative analysis of the cGMP concentration from cells expressing the PDE6 holoenzyme (α, β and γ) alone, and in the presence of w/t AIPL1 or AIPL1 variants as indicated. Independent assays were performed three or more times with samples analysed in triplicate in each assay. All AIPL1 variants showed statistically significant differences (*p* ≤ 0.01) with respect to w/t AIPL1 with the exception of the variants demarcated by NS (*p* > 0.01).

AIPL1 acts as a specific retinal co-chaperone that interacts with the molecular chaperone HSP90^4,5^. Therefore, we investigated the ability of the AIPL1 variants to bind recombinant HSP90α and HSP90β by performing quantitative ELISA experiments (Fig. 6a). The mean of the absorbance at 450 nm of each AIPL1 variant was normalized to the level of expression in the cell lysate and the results in Fig. 6a are calculated relative to w/t AIPL1. AIPL1 variants in the FKBP-like domain are shown as dark grey bars, in the TPR domain as white bars, in the region between the TPR and PRD as striped bars and in the PRD as light grey bars. The AIPL1 variants p.T39N, p.W72R, p.C89Y, p.Y194X, p.S198F, p.I206N, p.W222X, p.E245X, p.R270H, p.L293P, p.E309Dins, p.A364G and p.P376S had a statistically significant reduction of the interaction with both isoforms of HSP90 (α and β) compared with w/t AIPL1, while p.G122R, p.H130Q, p.Q298H, p.A352_P355del, p.P366R, p.E369_T372dup and p.A371_P374dup are not compromised in binding. AIPL1 variants affecting the TPR domain, including the missense mutants p.S198F and p.R270H and the premature translation termination mutants p.Y194X, p.W222X and p.E245X, had the greatest impact on HSP90 interaction as expected. Next, we investigated the impact of the AIPL1 variants on PDE6 activity using heterologous expression of functional rod PDE6 in HEK293T cells (Fig. 6b). We conducted our assays in the presence of the two catalytic subunits (PDE6α and PDE6β) and the regulatory subunit PDE6γ as described previously in Sacristan-Reviriego et al.^5^. Using a cGMP ELISA assay, we show that the PDE6 holoenzyme alone increased intracellular cGMP levels above background, and that the co-expression of AIPL1 with the PDE6 holoenzyme significantly increased the cGMP levels above the basal PDE6 levels. Analysis of the AIPL1 variants revealed that the p.T39N, p.W72R and p.C89Y variants in the FKBP-like domain had the greatest impact on cGMP levels and were completely unable to modulate these levels above PDE6 basal levels, whilst the impact of the FKBP-like domain variants p.G122R and p.H130Q was less significant. Interestingly, all the variants in the TPR domain and the p.E309Dins between the TPR domain and PRD also had an impact on cGMP levels, with the p.R270H variant unable to modulate cGMP level above basal levels. The variant p.Q298H between the TPR domain and PRD was as efficient as w/t AIPL1 in modulating cGMP levels. Within the PRD, none of the variants investigated led to a deficit in the modulation of cGMP levels compared to w/t AIPL1.

### The FKBP and the TPR domains of AIPL1 are essential for its functional activity

**Figure 7:**
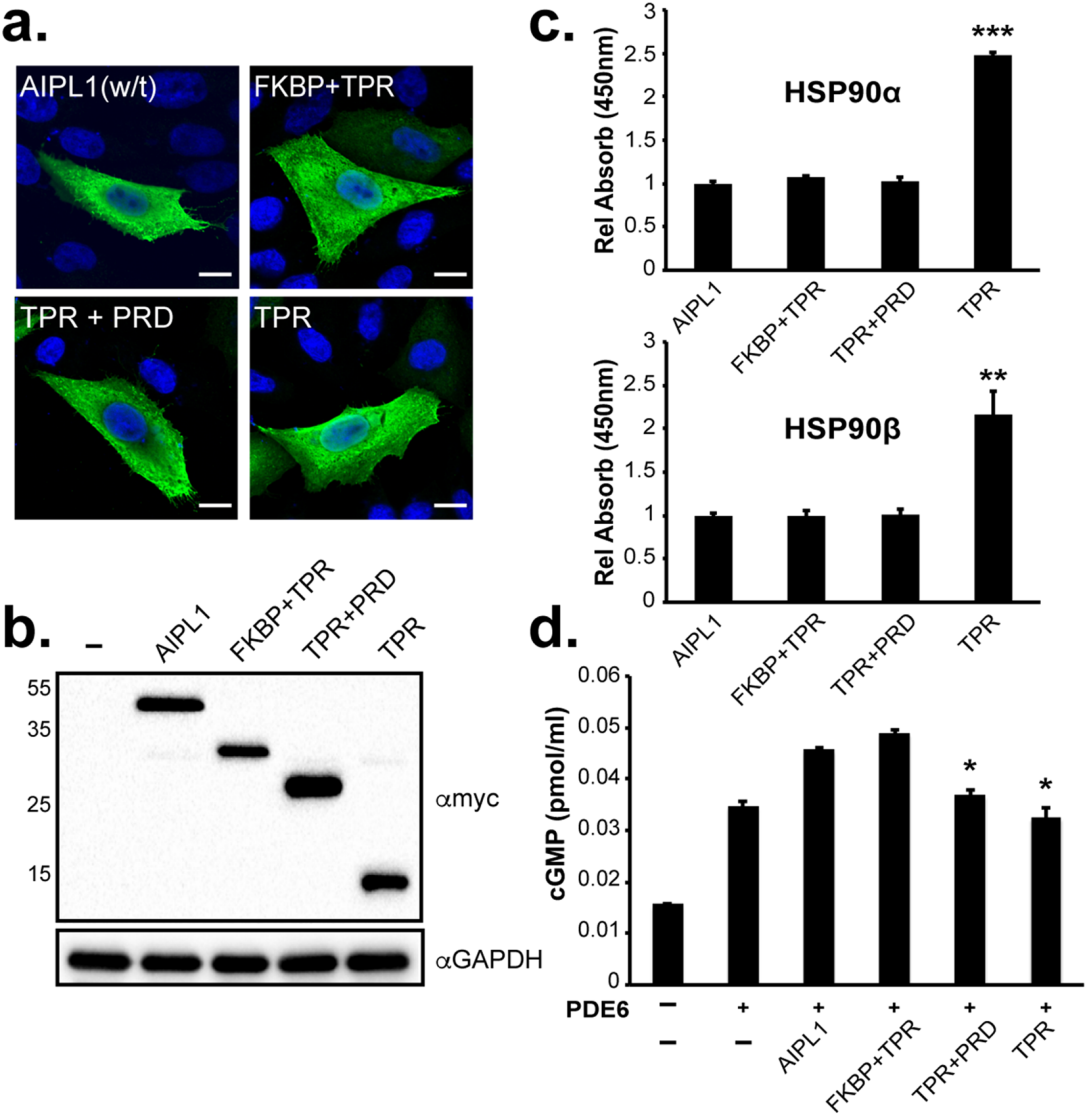
Functional characterization of AIPL1 domains. Subcellular localization and expression of AIPL1 domains. (**a**) Indirect immunofluorescent confocal microscopy and (**b**) western blotting analysis using anti-myc antibody. (**c**) Quantitative ELISA analysis of the interaction of AIPL1 domains with purified human recombinant HSP90α and HSP90β. The absorbance of each interaction was normalized to the expression level detected by western-blotting and plotted relative to w/t AIPL1 = 1.0. (**d**) Quantitative analysis of the cGMP concentration from cells expressing the PDE6 holoenzyme (α, β and γ) in the presence of AIPL1 domains. Assays were performed in triplicates. Statistically significant differences with respect to w/t AIPL1 are indicated by asterisks (*p < 0.05, **p < 0.01 and ***p < 0.001).

The role of the PRD and its contribution to AIPL1-associated pathogenesis are poorly understood. Therefore, we examined how the lack of the PRD domain, the FKBP-like domain or both the FKBP-like and PRD affect the ability of AIPL1 to bind HSP90 and promote the proper assembly and activity of PDE6. For that purpose, we engineered three different constructs: p.S328X (ΔPRD) including the FKBP-like and TPR domains (FKBP + TPR), p.M1_N168del (ΔFKBP) including the TPR domain and PRD (TPR + PRD) and p.N168-S328X (ΔFKBP + ΔPRD) including only the TPR domain (TPR). Immunofluorescent confocal microscopy (Fig. 7a) and western blotting (Fig. 7b) of transfected cells showed a normal cellular distribution and expression of all three constructs, as compared to w/t AIPL1. Quantitative ELISA experiments revealed that the lack of the PRD or FKBP domain did not reduce the binding of AIPL1 with recombinant HSP90α and HSP90β (Fig. 7c). Interestingly, the TPR domain alone interacted significantly better with HSP90α/β, showing a two-fold increase over w/t AIPL1. We further investigated the impact of the AIPL1 domains on cGMP levels following the heterologous expression of rod PDE6 (Fig. 7d). The lack of the PRD domain did not have any effect on PDE6 activity therefore this domain appears to be completely dispensable for this function (Fig. 7d) as well as for the interaction with HSP90 (Fig. 7c). On the contrary, the expression of TPR + PRD or TPR alone could not restore cGMP levels to that seen with full length w/t AIPL1, confirming that the FKBP domain is absolutely essential.

## Discussion

**Figure 8:**
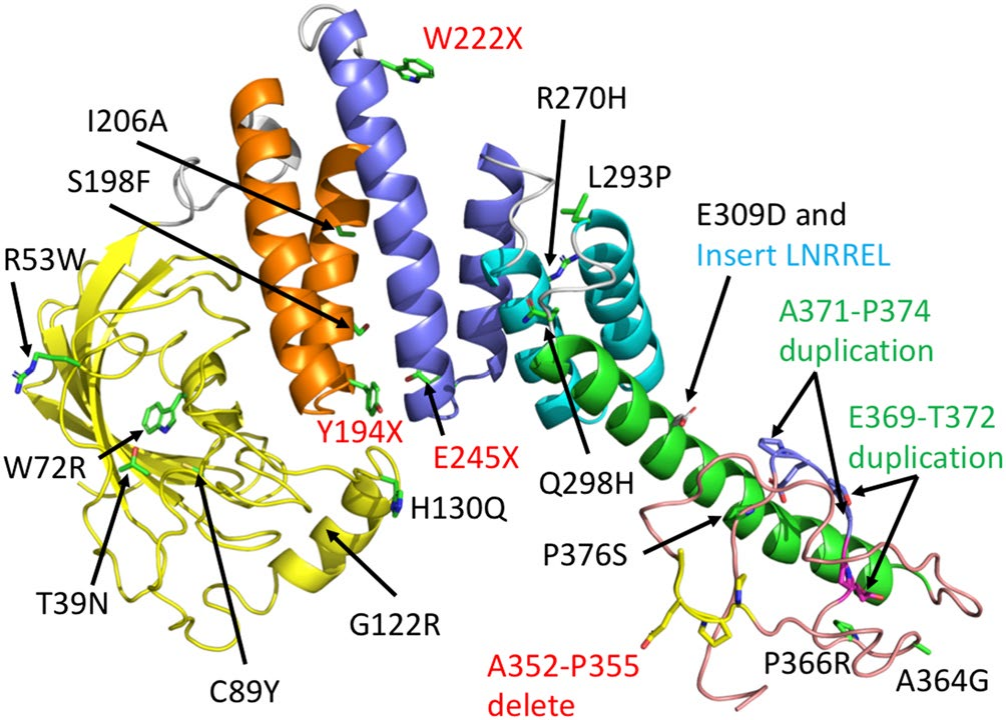
PyMol cartoon of an AIPL1 phyre2 model showing the location of mutations. The domains of AIPL1 are coloured as FKBP-like domain (yellow), TPR1 (gold), TPR2 (blue), TPR3 (cyan), helix 7 of the TPR domain (green) and PRD (salmon). Linkers between domains are shown in grey. Mutations that involve a C-terminal truncation or deletion are also indicated in red text, insertions in cyan text and duplications in green text.

This study reports the functional assessment of bi- or monoallelic *AIPL1* sequence variants, associated with a broad range of retinal diseases, including autosomal recessive LCA and RP, and autosomal dominant CORD and RP. *AIPL1* sequence variants investigated include missense and nonsense variations, as well as small insertions, duplications and deletions in the coding sequence (Fig. 8).

With the exception of p.G122R and p.H130Q, our data show that all variants in the FKBP-like domain (p.T39N, p.W72R, p.C89Y) (Fig. 8) and in the TPR domain (p.Y194X, p.S198F, p.I206A, p.W222X, p.E245X, p.R270H, p.L293P) (Fig. 8) are defective both in their ability to mediate an interaction with HSP90 and to modulate cGMP levels. We therefore confirm that these variants are disease-causing in accordance with all available evidence, including their allele frequency, in silico predictions of pathogenicity, and association with severe disease (LCA) in patients. We also report a novel disease-causing variant in *AIPL1*, c.666G>A (p.W222X), causing severe retinal degeneration (Fig. 1) and early loss of vision. Notably, the p.Y194X, p.S198F, p.W222X, p.E245X, and p.R270H variants in the TPR domain had the greatest impact on the interaction with HSP90, whilst p.T39N, p.W72R and p.C89Y in the FKBP-like domain had the greatest impact on the modulation of cGMP levels. However, sequence variants in the TPR domain also had an impact on the modulation of cGMP levels, and reciprocally, variants in the FKBP-like domain had an impact on HSP90 interaction. Our functional assays thus show that the FKBP-like and TPR domains are important for both HSP90 interaction and the modulation of cGMP levels. This confirms our previously reported conclusions that the relative conformational organization and orientation of these domains is important and necessary for AIPL1 function^5^.

Within the linker between the TPR domain and PRD, the c.894G>C (p.Q298H) sequence variant has been reported as a rare heterozygous change in a single patient with no second allele identified by direct sequencing of the *AIPL1* gene^39^. The in silico predictions of p.Q298H pathogenicity are inconclusive, and in this study, p.Q298H was consistently comparable to wild type AIPL1 in all assays, with no deficit in HSP90 binding or cGMP modulation. We therefore conclude that c.894G>C (p.Q298H) is a rare benign polymorphism. This is supported by the fact that p.Q298H occurs in the loop connecting the 6th and 7th α-helix of the TPR domain and it is solvent exposed (Fig. 8). In contrast, p.E309DinsLNRREL had a significant impact on both HSP90 interaction and cGMP modulation. The c.926_927insCCTGAACCGCAGGGAGCT (p.E309DinsLNRREL) sequence variant was reported as a homozygous change in an LCA patient with undetectable ERG responses and significantly attenuated visual acuity (hand motion)^37^, altogether confirming the disease-causing status and association of this variation with LCA. The insertion maps to the distal end of the 7th α helix of the TPR domain^44^ (Fig. 8), and is expected to considerably disrupt the topology of this domain.

The variants p.G122R and p.H130Q in the FKBP-like domain are unique amongst the variants investigated in this study, in that while they had no impact on HSP90 interaction, they were significantly compromised with respect to the modulation of cGMP levels. The p.G122R variant has been reported in association with the milder phenotypes of late onset retinal degeneration or RP. In this study, we report a pair of siblings that are compound heterozygous for c.364G>C (p.G122R) and c.834G>A (p.W278X) diagnosed with mild RP. Similarly, Jacobson et al.^25^ reported a patient with the same biallelic *AIPL1* variants who was first evaluated at the age of 45 years, presenting with widespread later onset retinal degeneration and macular involvement. In addition, we report a patient that presented with RP at the age of 34 years, who was homozygous for a different nucleotide change (c.364G>A) coding for the same p.G122R variant. This c.364G>A (p.G122R) sequence variant has also been reported in a patient compound heterozygous for c.834G>A (p.W278X)^34^; who presented at the age of 79 years with an extinguished ERG and visual acuity of hand motion in both eyes. Collectively, the clinical data combined with our functional assays suggest that the p.G122R variant is associated with a milder later-onset but progressive form of retinal degeneration, and may thereby behave as a rare hypomorphic allele. Hypomorphic variants in other genes including *RPE65, CEP290*, *NMNAT1* and *ABCA4* have also recently been associated with milder forms of inherited retinal disease with slower progression (later onset)^45–49^. Similar to p.G122R, the variant p.H130Q had no effect on HSP90 binding but was significantly compromised with respect to cGMP modulation. Whilst detailed clinical data is absent for the patient harbouring this variant, our data suggest that p.H130Q, like p.G122R, may behave as a rare hypomorphic variant. Both p.G122R and p.H130Q are located within an insert region between residues 90 and leucine 146 unique to the FKBP-like domain of AIP and AIPL1 that may act as a flexible lid to occlude the ligand-binding hydrophobic cavity^17,19,20,50^ (Fig. 8). The insert region of AIPL1 is well structured and comprised of three α helices (α2, α3, α4)^19^. G122 and H130 are located in the middle and immediately distal to the α3 helix in the insert region (Fig. 8). Deletion of residues 111 to 132 (encompassing G122 and H130) in the insert region had no structural impact on the FKBP-like domain, but completely abolished the interaction with an isoprenyl group due to the loss of extensive α2 residue side-chain contributions to ligand binding^17,19^. Interestingly, molecular dynamics simulations revealed α3 to be the most flexible region of the FKBP-like domain, favouring occlusion to the entrance of the hydrophobic ligand-binding cavity in a dynamic and intermittent fashion^19^. We propose therefore, that p.G122R and p.H130Q do not contribute to, or significantly impact, the binding of the isoprenyl group, but may alter the conformation of the lid and accessibility to the hydrophobic ligand binding cavity, thus modulating, but not severely impacting AIPL1 function.

The variants within the primate-specific PRD of AIPL1 require special consideration, as the role of the PRD remains unresolved. The PRD of human AIPL1 is an extended and unstructured random coil that lacks secondary structure^18^ (Fig. 8). Previously, Li et al.^51^ reported that while the PRD did not impact the structural properties and thermal stability of AIPL1, deletion of the PRD enhanced the affinity of AIPL1 for full length HSP90, suggesting that the PRD may have an inhibitory effect on HSP90 binding. Our data also show that the greatest HSP90 binding efficiency is achieved with the TPR domain alone. However, compared to full length wild-type AIPL1, we show that the PRD is completely dispensable for both the interaction with HSP90 and the modulation of PDE6 activity. Similarly, deletion of the PRD domain has been shown to have no effect on the interaction of AIPL1 with the HSP90 C-terminal peptide TSRMEVEED or the interaction of AIPL1 with a farnesylated and carboxymethylated C-terminal PDE6α peptide^18^. In accordance with these data, we show that all the variants investigated in the PRD domain (p.A352_P355del; p.A364G; p.P366R; p.E369_T372dup; p.A371_P374dup; p.P376S) had either a minimal or no significant effect on binding HSP90, and did not compromise the modulation of cGMP levels, pointing to these variants either having no role in disease or contributing to pathogenesis via a different and as of yet uncharacterised mechanism.

In patients, the PRD variants, with the exception of c.1126C>T (p.P376S), have been identified as monoallelic variants in association with a range of different clinical phenotypes, including LCA, EOSRD, adCORD, early-onset RP and RP. Indeed, c.1126C>T (p.P376S) has been reported in patients as a homozygous, compound heterozygous or heterozygous change associated with LCA, EOSRD, or RP. Interestingly, in all reported patients that are compound heterozygous for c.1126C>T (p.P376S), the other sequence variant is invariably c.341C>T (p.T114I) in these patients, and the maternal *cis*-allelic inheritance of c.1126C>T (p.P376S) and c.341C>T (p.T114I) has been reported in one patient. This suggests that c.1126C>T (p.P376S) and c.341C>T (p.T114I) are a common haplotype, the inheritance of which exceeds the frequency with which one would expect these variants to occur together in a rare autosomal recessive disease. Indeed, the allele frequency of c.1126C>T (p.P376S) is higher than expected with 27 and 51 homozygotes detected in ExAC and gnomAD respectively. This variant has also been shown not to segregate with disease^41^. Our results show that p.P376S has no impact on cGMP levels in our functional assays, and we conclude that this variation is not associated with disease in these patients. Similarly, we conclude that the PRD variants c.1091C>G (p.A364G), c.1097C>G (p.P366R), c.1103_1114dup (p.E369_T372dup) and c.1111_1122dup (p.A371_P374dup), reported as monoallelic variants in patients, are unlikely to be the cause of disease in these patients. The c.1103_1114dup (p.E369_T372dup) and c.1111_1122dup (p.A371_P374dup) duplications translate to an identical product that does not result in the addition or loss of any proline-rich motifs (PRM), and may thus not significantly impact the repetitive unstructured PRD.

Finally, the PRD variant c.1053_1064delTGCAGAGCCACC (p.A352_P355del) (Fig. 8) has been reported to cause adCORD or early-onset RP^26^. The pathogenesis of this variant was investigated in transgenic mouse models in which the wild-type or the c.1053_1064delTGCAGAGCCACC (p.A352_P355del) human *AIPL1* transgene under the control of the mouse cone/rod *Crx* promoter was expressed in an *Aipl1* null background^52^. In single transgenic mice, the mutant transgene led to a CORD phenotype, predominantly leading to a slow and progressive cone degeneration with near complete loss of cones at P125, and early photopic defects preceding cone loss. Progressive rod photoreceptor degeneration was considerably delayed compared to cone degeneration and stabilised after P90. Decreased levels of rod PDE6α, rod PDE6β, PDE6γ and cone PDE6α’ were observed in these animals at P16 before the onset of cone and rod degeneration. In double transgenic mice, the p.A352_P355del was shown to have a dominant negative effect on wild-type human AIPL1, but not on wild-type murine Aipl1, which was able to rescue the CORD phenotype, confirming that the dominant negative effect of p.A352_P355del is mediated through the PRD. However, this study also showed that the pathogenesis mediated by this disease-causing variant differs in rods and cones. An ~ 40–50 fold excess of AAV-delivered wild type human AIPL1 relative to p.A352_P355del AIPL1 rescued cone-mediated photopic vision and restored the levels of cone PDE6α’. However, the excess levels of human AIPL1 did not restore rod-mediated scotopic vision, and while the near statistically relevant rescue of PDE6α and PDE6β were achieved, no rescue of PDE6γ was achieved. Thus, the deficit in PDE6γ occurs independently of either the presence or absence of wild-type AIPL1, pointing to a dominant gain-of-function in these cells. This pathogenic effect might be mediated via as of yet unidentified photoreceptor-specific interacting protein(s). Our in vitro functional assays showed that p.A352_P355del did not lead to a significant defect in either HSP90 interaction or cGMP modulation. However, our assay entails the heterologous reconstitution of rod PDE6α, PDE6β and PDE6γ in the absence of photoreceptor-specific proteins, and we conclude that while our assay is useful for the functional validation of loss-of-function mutations, it is not suitable for the validation of dominant disease involving a gain-of-function mechanism. To date, the c.1053_1064delTGCAGAGCCACC (p.A352_P355del) variant is the only *AIPL1* sequence variant associated with a dominantly inherited phenotype. The other PRD variants investigated in this study are all associated with autosomal recessive loss-of-function disease, and we conclude from our assays that these variants are not disease-associated.

In summary, we confirm the disease-causing status of sequence variants in the FKBP-like and TPR domains of AIPL1, as well as within the linker between these domains, and confirm that p.Q298H is a rare benign variant. We also show that certain variants (p.G122R) may behave as rare hypomorphic alleles, thus improving the prospects of timely therapeutic intervention for these patients. Finally, we show that the PRD of AIPL1 is dispensable for both HSP90 interaction and PDE6 activity, and that variants in this domain do not lead to deficits in the modulation of cGMP levels. We confirm that the PRD variants investigated do not cause disease, with the exception of p.A352_P355del associated with adCORD, which causes pathogenesis via alternative PRD-mediated dominant negative and gain-of-function mechanisms mediated by this domain.

## Materials and methods

### *AIPL1 sequence variations, nomenclature and *in silico* analysis*

In this study, we investigated 21 molecularly confirmed homozygous, compound heterozygous or heterozygous *AIPL1* variations (missense, nonsense, insertions, duplications or deletions) in new and previously reported cases of patients with retinal degeneration. The *AIPL1* coding sequence is numbered according to the Ensembl Transcript ENST00000381129 (RefSeq NM_014336, NP_055151) where the cDNA uses + 1 as the ATG translation initiation codon, with the initiation codon designated as codon 1. Nomenclature of *AIPL1* sequence variations followed HGVS (Human Genome Variation Society, in the public domain https://varnomen.hgvs.org) standards. *AIPL1* variations were investigated using two in silico software prediction programmes: SIFT (Sorting Intolerant From Tolerant; in the public domain, https://sift.jcvi.org) and PolyPhen-2 (Polymorphism Phenotyping v2; in the public domain, https://genetics.bwh.harvard.edu/pph/index.html). Phyre2 was used to build a working model of the AIPL1 protein^53^ and the model was visualised with the PyMol (Schrödinger Inc, USA).

### DNA manipulation and plasmids

General DNA methods were performed using standard techniques. For cloning and amplification of plasmid DNA, the *Escherichia coli* strain DH5α (*fhuA2 Δ(argF-lacZ)U169 phoA glnV44 Φ80 Δ(lacZ)M15 gyrA96 recA1 relA1 endA1 thi-1 hsdR17*) from New England Biolabs (NEB) was used. Expression of myc-tagged AIPL1 was carried out using pCMV-Tag3C-*AIPL1* plasmid^54^. *AIPL1* mutagenesis was undertaken by PCR site-directed mutagenesis (SDM) using the Q5 Site-Directed Mutagenesis Kit (NEB). Primers were designed using NEBaseChanger software (in the public domain, https://nebasechanger.neb.com), and SDM PCRs were conducted according to suggested conditions. *AIPL1* sequences were checked by Sanger sequencing (Source Bioscience). To express rod PDE6 subunits in cells, pcDNA3.1AXpress-PDE6α, pCMV-HA-PDE6β and pCMV-Myc-PDE6γ were used^5^.

### Cell culture and transfection

Dulbecco’s modified Eagle’s medium DMEM (Invitrogen) with 10% heat inactivated fetal bovine serum (FBS), 100 units/ml of penicillin and 100 µg/ml of streptomycin was used to culture Chinese Hamster Ovary (CHO) and HEK293T (human embryonic kidney) cells with an atmosphere of 6% CO_2_ at 37 °C. For immunofluorescence, cells were seeded into eight-well chamber slides (3.5 × 10^4^ cells per well). In order to obtain protein extracts, cells were seeded into six-well plates (5 × 10^5^ cells per well). Plasmids were transfected using TransIT-LT1 transfection reagent according to the manufacturer’s instructions (Mirus).

### Immunocytochemistry

24 h after transfection, CHO cells were washed twice with phosphate buffered saline (PBS), then fixed with 4% paraformaldehyde for 10 min and permeabilized in 0.5% triton X-100 for another 10 min. Blocking was performed using 3% BSA, 10% goat serum in PBS for 1 h at room temperature. A 1h incubation with primary antibody mouse monoclonal anti-myc (1:1000) (clone 9E10, Sigma) was followed by 3 washes with PBS and a 1h incubation with secondary donkey anti-mouse Alexa 488 antibody (1:600) (ThermoFisher Scientific). After 3 washes with PBS, cells were incubated with DAPI (2 mg/ml) for 5 min, mounted in fluorescent mounting medium (Dako) and visualized with a Zeiss LSM710 laser scanning confocal microscope. The images were exported from LSM Browser and processed using ImageJ and Adobe Photoshop.

### Cells extracts and immunoblotting

Transfected cells were washed in cold PBS and lysed on ice in RIPA buffer (50 mM Tris HCl pH 7.5, 150 mM NaCl, 1 mM EDTA, 1% NP-40, 0.5% sodium deoxycholate, 0.1% SDS) with 2% protease inhibitor cocktail (Sigma). Cells were scraped, sonicated and centrifuged at 13,000 rpm for 30 min at 4 °C. The supernatant was collected and determination of the protein concentration was carried out using the BCA assay (ThermoScientific). Protein extracts were resolved by denaturing SDS-PAGE and transferred onto nitrocellulose membranes. Mouse monoclonal anti-myc (1:1000) (clone 9E10, Sigma) and rabbit polyclonal antisera anti-AIPL1 (1:1000) (Ab-hAIPL1)^1^ antibodies ware used to immunodetect AIPL1 protein variants. GAPDH inmunodetection using mouse monoclonal anti-GAPDH (1:30,000) (Sigma) served as a loading control. Goat anti-mouse (1:30,000) and anti-rabbit (1:30,000) secondary antibodies conjugated with horseradish peroxidase were from Pierce Biotechnology. Chemiluminescent detection was performed using the Luminata Crescendo (Millipore) reagent. Western blot densitometry was carried out using ImageJ.

### HSP90 ELISA binding assay

Assays were performed as described in Sacristan-Reviriego et al.^5^. Briefly, purified HSP90α or HSP90β (80 nM) were incubated in Immulon 4HBX 96-well plates (Fisher Scientific) for at least 1 h at 4 °C. 96-well plates were blocked using 1% blocking reagent (Sigma) in the same buffer (100 mM NaHCO_3_ pH 8.5) for 1 h and then washed 3 times with chilled TBST (50 mM Tris HCl pH 7.5, 150 mM NaCl, 0.075% Tween-20) to remove excess protein. Cell lysates from transfected cells with wild type (w/t) AIPL1 or AIPL1 variants were prepared as described above and added to the wells in lysis buffer (20 mM Tris HCl pH 7.5, 100 mM NaCl, 5 mM MgCl_2_, 0.075% Tween-20) containing 2% protease inhibitor cocktail. After a 1 h incubation at 4 °C, wells were washed 5 times with chilled lysis buffer. Incubation with mouse anti-myc antibody (1:1000) for 1 h was followed by 5 washes and another 1 h incubation with anti-mouse HRP conjugated antibody (1:10,000). After 5 final washes, the plate was incubated with substrate 3,3′,5,5′-tetramethylbenzidine (TMB) (Sigma) for colorimetric detection at 30 °C for 30 min. The reaction was stopped using 0.5 M H_2_SO_4_ and the absorbance measured at 450 nm. For comparison of the AIPL1 variants to w/t AIPL1, the absorbance measured at 450 nm was normalised to the expression level in cell lysates detected by SDS-PAGE and western blotting.

### PDE6 activity assay

Determination of intracellular cGMP concentration from acetylated samples was carried out using the Cyclic GMP ELISA Kit (Cayman Chemical) as described in Sacristan-Reviriego et al.^5^. In brief, cells were lysed in 0.1 M H Cl at room temperature and the supernatant was obtained after centrifugation at 3000 rpm for 10 min. The assays were done in triplicate and repeated at least three times.

### Statistical analysis

Statistical analyses to determine *p* values for intergroup comparisons were conducted using the unpaired Student *t*-test. *p* > 0.01 was considered not significant (NS).

### Ethics

All procedures were adherent to the tenets of the Declaration of Helsinki and carried out with approved protocols of Moorfields Eye Hospital and the Montpellier University Hospital. All procedures were approved by Moorfields Eye Hospital Ethics Committee and the Ministry of Public Health under the authorization number 11018S, and were performed with written informed consent from patients or legal guardians in the case of children.

## Supplementary information

Supplementary Information.

## Data Availability

Data produced and analysed in this study are available from the corresponding author on request.
