# Supplementary material for: Clinical and functional analyses of AIPL1 variants reveal mechanisms of pathogenicity linked to different forms of retinal degeneration
Source: Sci Rep. 2020 Oct 16;10:17520. doi: 10.1038/s41598-020-74516-9 (PMC7567831; doi:10.1038/s41598-020-74516-9)
Supplement: Supplementary file 1 — Supplementary Information. [file 41598_2020_74516_MOESM1_ESM.pdf]

Supplementary data

Clinical and functional analyses of *AIPL1* variants reveal mechanisms of pathogenicity linked to different forms of retinal degeneration

Almudena Sacristan-Reviriego[1], Hoang Mai Le[1], Michalis Georgiou[1,2], Isabelle Meunier[3], Beatrice Bocquet[3], Anne-Françoise Roux[4], Chrisostomos Prodromou[5], James Bainbridge[1,2], Michel Michaelides[1,2] and Jacqueline van der Spuy[1*]

[1]UCL Institute of Ophthalmology, University College London, 11 – 43 Bath Street, London, EC1V 9EL, UK

[2]Moorfields Eye Hospital, City Road, London, EC1V 2PD, UK

[3]Centre National de Référence Maladies Sensorielles Génétiques. Service Ophtalmologie Hôpital Gui de Chauliac - CHRU de Montpellier. 80 rue Augustin Fliche – 34295 Montpellier, France

[4]Laboratoire de Génétique Moléculaire, CHU de Montpellier, Université de Montpellier, Montpellier, France.

[5]Biochemistry and Biomedicine, University of Sussex, Brighton, Falmer, BN1 9QG, UK

*Corresponding author: j.spuy@ucl.ac.uk

Supplementary Table 1: Clinical information of patients with *AIPL1* variations

Case	Allele 1	Allele 2	Visual Acuity R - L	OCT	Posterior Pole Findings	ERG	Clinical Diagnosis	Reference
**Patients with *AIPL1* variations identified on both alleles**								
**P1**	c.116C>A; **p.T39N**	c.116C>A; **p.T39N**	N/A	N/A	Macular degeneration, diffuse pigmentary retinopathy	N/A	LCA	46
**P2**	c.214T>C; **p.W72R**	c.265T>C; p.C89R	PL - PL	Abnormal lamination, foveal depression, reduced foveal retinal thickness, foveal ONL barely discernible or not detectable	N/A	Undetectable	LCA	25
**P3**	c.266G>A; **p.C89Y**	c.266G>A; **p.C89Y**	N/A	N/A	N/A	Markedly diminished	LCA	47
**P4**	c.364G>A; **p.G122R**	c.834G>A; p.W278X	HM - HM	N/A	Macular dystrophy	Undetectable	LCA	32
**P5**	c.364G>A; **p.G122R**	c.364G>A; **p.G122R**	20/50 – 20/100	N/A	N/A	Discernible rod and cone responses	RP	This study
**P6**	c.364G>C; **p.G122R**	c.834G>A; p.W278X	20/60 - 20/400	Deep foveal depression, loss of foveal ONL, signs of retinal remodeling in the macular region	Bull's-eye – like lesion in the foveal region, pigmentary retinal degeneration with atrophy extending from the arcades into the midperipheral retina and attenuated retinal vessels	Undetectable	RP/ Late onset retinal degeneration	25
**P7**	c.364G>C; **p.G122R**	c.834G>A; p.W278X	20/20 - 20/20	Extensive loss of the photoreceptor-RPE complex and the EZ, EZ and IZ are only visible in the foveal zone	N/A	Dark-adapted and light adapted full-field ERG responses are reduced within 50% and 25% respectively of the normal range	Mild RP	This study
**P8**	c.364G>C; **p.G122R**	c.834G>A; p.W278X	20/20 - 20/20	The photoreceptor-RPE complex and the EZ are only visible in the foveal zone	N/A	N/A	Mild RP	This study

**P9**	c.582C>G; **p.Y194X**	c.834G>A; p.W278X	PL - PL	N/A	Pigmentary changes	N/A	LCA	48
**P10***	c.666G>A; **p.W222X***	c.834G>A; p.W278X	HM/30cm - HM/30cm	Loss of outer retinal structure	N/A	N/A	LCA	This study
**P11**	c.733G>T; **p.E245X**	c.834G>A; p.W278X	6/240 - 6/240	N/A	Pigmentary changes	N/A	LCA	48
**P12**	c.809G>A; **p.R270H**	c.834G>A; p.W278X	HM - HM	N/A	Macular atrophy	Undetectable	LCA	49
**P13**	c.809G>A; **p.R270H**	c.834G>A; p.W278X	20/1200 – 20/1000	101/111, PSJ not preserved	Macular dystrophy	Undetectable	LCA	32
**P14**	c.809G>A; **p.R270H**	c.834G>A; p.W278X	N/A	N/A	N/A	Markedly diminished	LCA	47
**P15**	c.926_927ins CCTGAACCGCAGGGAGCT; **p.E309DinsLNRREL**	c.926_927ins CCTGAACCGCAGGGAGCT; **p.E309DInsLNRREL**	HM/50cm - HM/45cm	N/A	Attenuated vessels, carpet like retinal degeneration	Undetectable	LCA	36
**P16**	c.1126C>T; **p.P376S**	c.341C>T; p.T114I	N/A	N/A	N/A	N/A	LCA	26
**P17**	c.1126C>T; **p.P376S**	c.341C>T; p.T114I	20/400	N/A	Peripheral retinal mottling, severe maculopathy, moderate pigmentary retinopathy	N/A	LCA	23
**P18**	c.1126C>T; **p.P376S**	c.341C>T; p.T114I	N/A	N/A	Severe pigmentary retinopathy	N/A	LCA	23
**P19**	c.1126C>T; **p.P376S**	c.341C>T; p.T114I	N/A	N/A	N/A	N/A	EOSRD LCA	50
**P20**	c.1126C>T; **p.P376S**	c.341C>T; p.T114I	6/19 - 6/48	Reduced retinal and foveal thickness	Moderate pigmentary changes. Bone spicule retinopathy, no obvious macular atrophy but some pigmentary changes seen in the macula	Full field and rod ERG responses reduced in R and L, subnormal and delayed cone 30 Hz flicker ERG in R and L	ESORD	35 This study
**P21**	c.1126C>T; **p.P376S**	c.1126C>T**; p.P376S**	6/60 - PL	N/A	Pigmentary retinopathy with severe clumping, severe macular atrophy	Reduced responses in R, undetectable in L	EOSRD	35 This study

Supplementary Table 1: Clinical information of patients (unrelated probands) with *AIPL1* variations - Continue

Case	Allele 1	Allele 2	Visual Acuity R - L	OCT	Posterior Pole Findings	ERG	Clinical Diagnosis	Reference
**Patients with a single *AIPL1* variation identified on one allele**								
**P22**	c.157C>T; **p.R53W**	N/A	N/A	N/A	N/A	N/A	LCA	51
**P23**	c.214T>C; **p.W72R**	N/A	N/A	N/A	N/A	N/A	LCA	33
**P24**	c.390C>A; **p.H130Q**	N/A	N/A	N/A	N/A	N/A	EOSRD<br>LCA	35
**P25**	c.593C>T; **p.S198F**	N/A	N/A	N/A	N/A	N/A	EOSRD<br>LCA	35
**P26**	c.617T>A; **p.I206N**	N/A	N/A	N/A	N/A	N/A	LCA	51
**P27**	c.878T>C, **p.L293P**	N/A	N/A	N/A	N/A	N/A	LCA	33
**P28**	c.894G>C; **p.Q298H**	N/A	N/A	N/A	N/A	N/A	EOSRD<br>LCA	35
**P29**	c.1053_1064del<br>TGCAGAGCCACC; **p.A352_P355del**	N/A	N/A	N/A	N/A	N/A	adCORD,<br>juvenile RP	26
**P30**	c.1091C>G; **p.A364G**	N/A	N/A	N/A	N/A	N/A	EOSRD<br>LCA	35
**P31**	c.1097C>G; **p.P366R**	N/A	N/A	N/A	N/A	N/A	EOSRD<br>LCA	35
**P32**	c.1103_1114dup; **p.E369_T372dup**	N/A	N/A	N/A	N/A	N/A	LCA	33
**P33**	c.1111_1122dup; **p.A371_P374dup**	N/A	20/200<br>(better eye)	N/A	Primarily peripapillary hypopigmentation with patchy pigment clumping as well as drusen like deposits and macular hypopigmentary changes	Undetectable/<br>Severely reduced	LCA	52
**P34**	c.1126C>T; **p.P376S**	N/A	N/A	N/A	N/A	N/A	LCA†	33
**P35**	c.1126C>T; **p.P376S**<br>c.341C>T; p.T114I<br>*Cis*-allelic inheritance from mother	N/A	N/A	N/A	N/A	N/A	LCA	34
**P36**	c.1126C>T; **p.P376S**	N/A	6/30 – 6/38	N/A	Moderate pigmentary changes, clumping<br>Macular pigmentation and atrophy	Absent rod and cone ERG responses, delayed 30 Hz flicker cone ERG of low amplitude.	EOSRD	35<br>This study
**P37**	c.1126C>T; **p.P376S**	N/A	3/60 - 3/60	N/A	Severe pigmentary retinopathy, no maculopathy	Reduced in R and L	RP<br>EOSRD	35<br>This study

R, right eye; L, left eye; HM, hand motion; PL, perception of light; RPE, retinal pigment epithelium; LCA, Leber congenital amaurosis; EOSRD, early-onset severe retinal dystrophy; RP, retinitis pigmentosa; adCORD; autosomal dominant cone-rod dystrophy; ONL, outer nuclear layer; N/A, not available; *, novel variation; †, did not segregate with disease. The AIPL1 variants investigated in this study are shown in **BOLD**.

Supplementary Figures

Figure Legends:

Supplementary Figure 1: (A) Full-field ERG from a 34-year-old man with a retinitis pigmentosa phenotype carrying a homozygous *AIPL1* p.G122R variant. Dark-adapted (rod responses) and light-adapted (cone responses) are reduced within 20% of the normal range. (B) Full-field ERG from an 18-year-old woman with a retinitis pigmentosa phenotype with *AIPL1* c.364G>C, p.G122R/c.834G>A, p.W278X variants. Dark-adapted (rod responses) are reduced within 50% and light-adapted (cone responses) within 25% of the normal range.

Supplementary Figure 2: Longitudinal infrared and autofluorescence imaging of the brother of the woman of the figure 2. (A-D) First examination at the age of 7 with nyctalopia noted at the age of 6. Visual acuity is 20/20 in both eyes. On infrared reflectance imaging, the vessels are preserved (A 30° field of view, C 50° field of view). On FAF imaging (B, D), there is a change of the autofluorescence of the macula and the nasal part of the optic nerve head with a hyperautofluorescence pattern in both eyes (white arrows). (E-J) Multimodal imaging of the brother at the age of 14: (E, G) infrared reflectance imaging, (F, H) FAF imaging, (I, J), SD OCT imaging. Visual acuity is 20/20. FAF imaging (F and H) discloses a macular hyperautofluorescent ring in both eyes. On SD-OCT (I and J), the PR+RPE complex and the EZ are only visible in the foveal zone (between blue arrows) in accordance with the visual acuity of 20/20.

Supplementary Figure 3: Full blots: Expression of AIPL1 variants. Western blotting analysis (different exposures) of wild-type (w/t) AIPL1 and the indicated AIPL1 variants. The proteins were resolved by SDS-PAGE on a 12% gel. (A) The anti-myc antibody recognizes the myc tag fused to the N-terminus of AIPL1. (B) The anti-AIPL1 antibody is directed against a C-terminal AIPL1 epitope [32]. GAPDH was detected as a loading control. * demarcates an *in silico* designed AIPL1 variant not found in patients and not associated with disease. (C) Relative quantitation of AIPL1 levels with the anti-myc antibody (upper panel) and the anti-AIPL1 antibody (lower panel).

Supplementary Figure 1

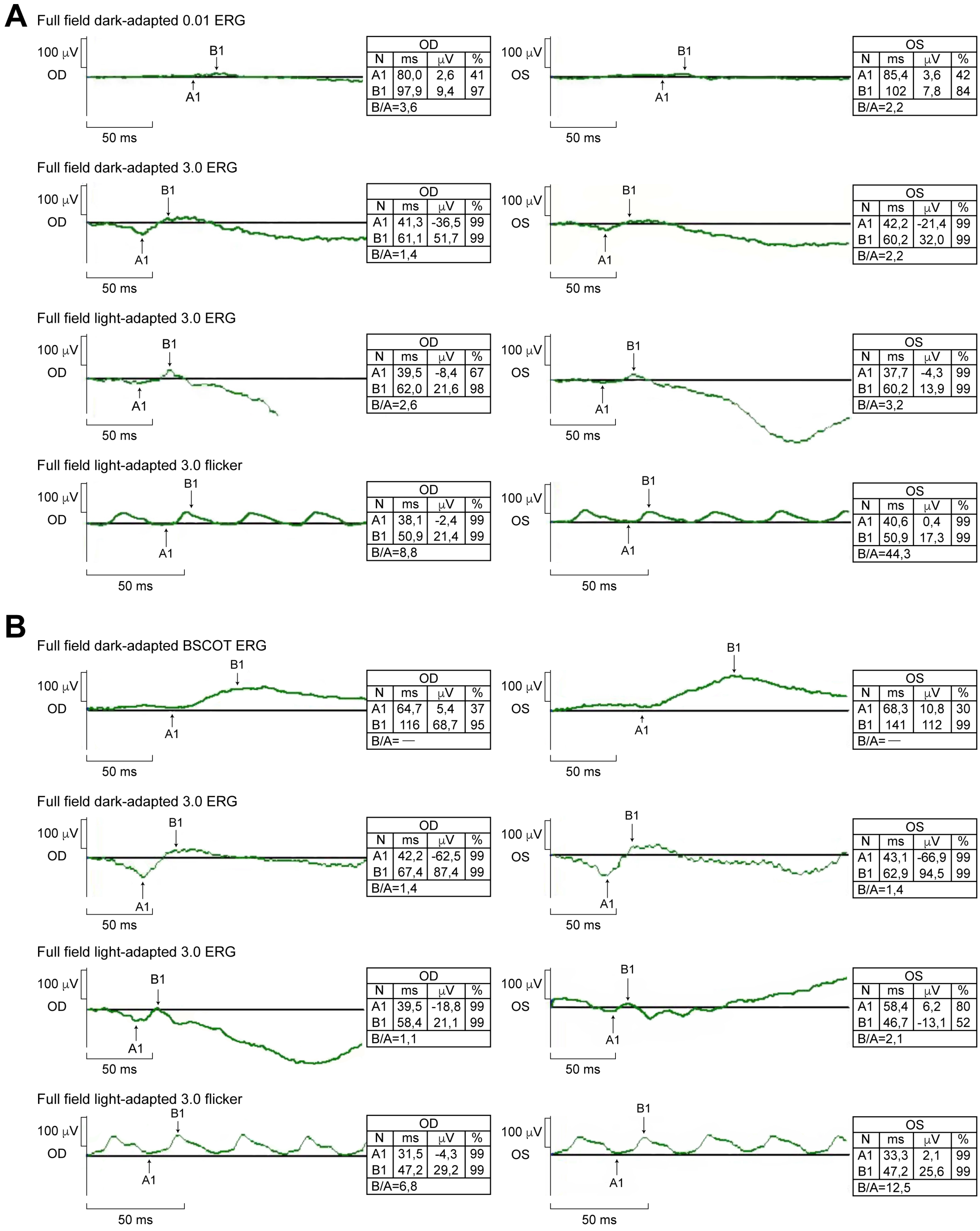

Supplementary Figure 2

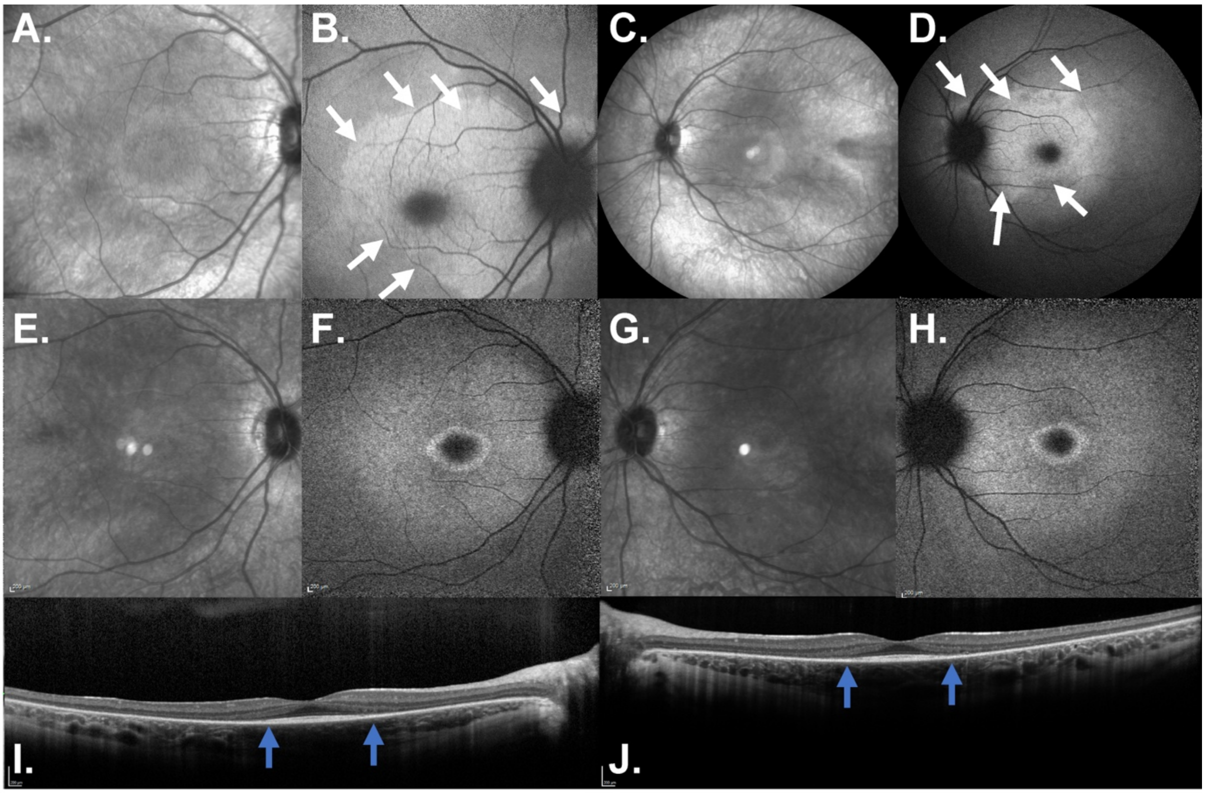

Supplementary Figure 3:

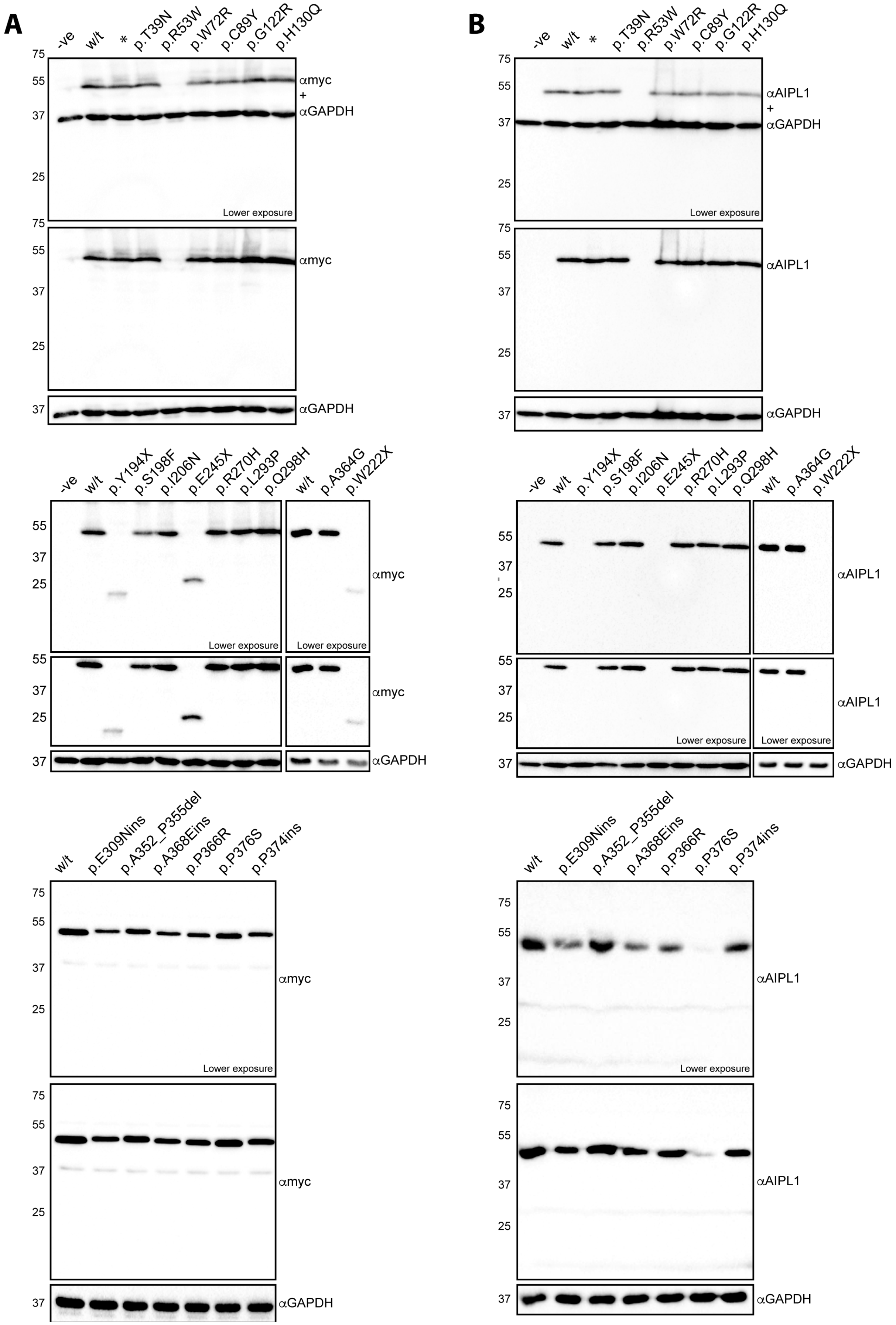

C

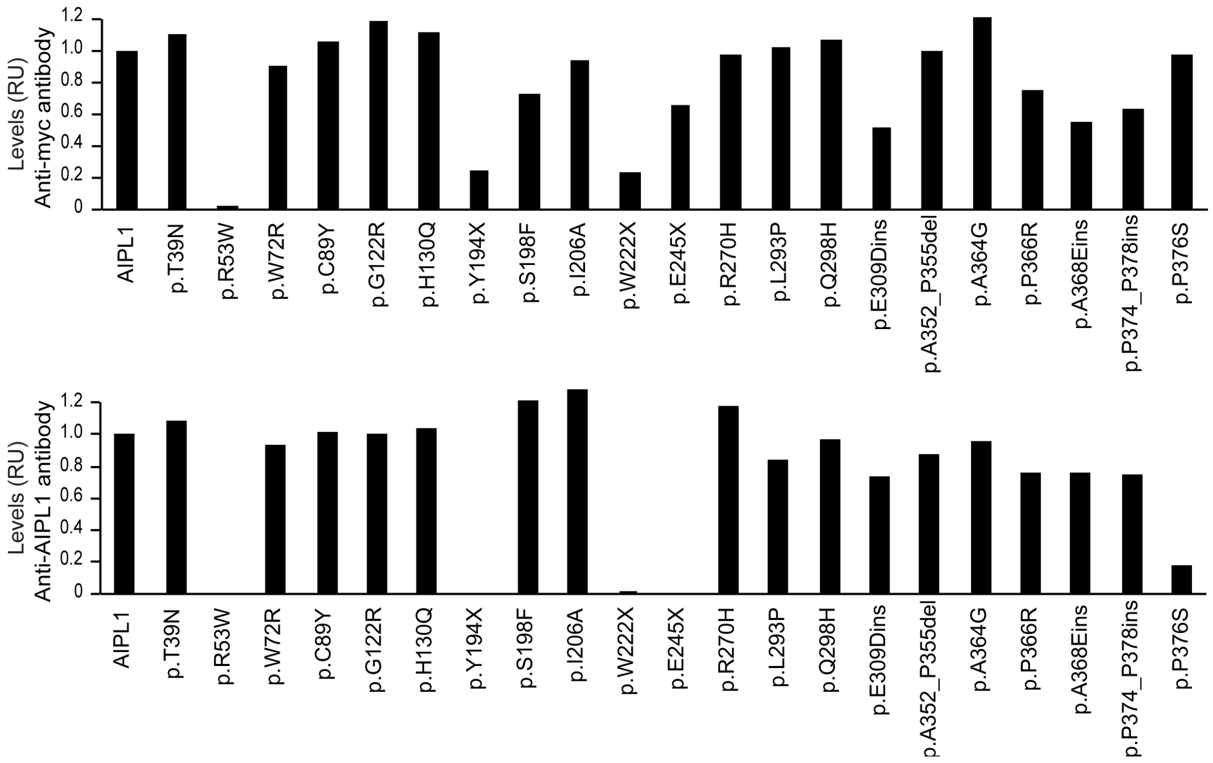
Levels (RU)
Anti-myc antibody
0
0.2
0.4
0.6
0.8
1.0
1.2
AIPL1
p.T39N
p.R53W
p.W72R
p.C89Y
p.G122R
p.H130Q
p.Y194X
p.S198F
p.I206A
p.W222X
p.E245X
p.R270H
p.L293P
p.Q298H
p.E309Dins
p.A352_P355del
p.A364G
p.P366R
p.A368Eins
p.P374_P378ins
p.P376S
Levels (RU)
Anti-AIPL1 antibody
0
0.2
0.4
0.6
0.8
1.0
1.2
AIPL1
p.T39N
p.R53W
p.W72R
p.C89Y
p.G122R
p.H130Q
p.Y194X
p.S198F
p.I206A
p.W222X
p.E245X
p.R270H
p.L293P
p.Q298H
p.E309Dins
p.A352_P355del
p.A364G
p.P366R
p.A368Eins
p.P374_P378ins
p.P376S
